# Research and development directions in vestibular rehabilitation: a bibliometric analysis

**DOI:** 10.3389/fresc.2026.1735847

**Published:** 2026-06-08

**Authors:** Chen Li, Ai-Song Guo, Jie-Ling Hu

**Affiliations:** 1 School of Nursing and Rehabilitation, Nantong University, Nantong, Jiangsu Province, China; 2 Department of Traditional Chinese Medicine, Affiliated Hospital of Nantong University, Nantong, Jiangsu Province, China; 3 Department of Neurology, Nantong Hospital of Traditional Chinese Medicine, Nantong, Jiangsu Province, China

**Keywords:** bibliometric, CiteSpace, dizziness, rehabilitation, vestibular, vestibular dysfunction, visualization analysis

## Abstract

**Background:**

Vestibular rehabilitation training (VRT) has become an indispensable part of treatment for dizziness disorders. VRT is an exercise method that can alleviate dizziness and improve gaze stability and postural control, mainly used for the treatment of patients with vestibular dysfunction.

**Methods:**

To analyse the current status, hot spots and trends in research on vestibular rehabilitation, data were collected from the Web of Science Core Collection and Scopus databases, and selected publications related to dizziness vestibular rehabilitation from 2014 to 2024. Visual analysis tools were utilized to build knowledge maps of the research field, analyzing the distribution of publications, authors, institutions, journals, collaboration networks, co-occurrence, clustering, and burst detection of keywords.

**Results:**

A total of 907 publications were included in this study, with an upward trend in the number of annual publications. The United States led in publication quantity, with the University of Pittsburgh ranking first among institutions. Professor Susan L. Whitney (University of Pittsburgh) was the most productive researcher. Recent research hotspots concentrated on unilateral vestibular hypofunction, vestibular neuronitis, and postural balance, providing clinical evidence for targeted VRT intervention.

**Conclusion:**

Research on VRT for dizziness is expanding progressively, with vestibular compensation and postural balance as core hotspots. The findings consolidate VRT's clinical application value in guiding personalized intervention for different vestibular disorders. Future research trends will focus on expanding VRT's application scope, formulating individualized protocols, and developing AI-aided targeted VRT, which will optimize clinical efficacy and promote the standardized development of this field.

## 1 Introduction

Peripheral vestibular disorders have garnered significant research interest due to their high prevalence, with acute unilateral vestibulopathy, formerly termed vestibular neuritis, ranking as the third most common acute peripheral vestibular condition, following benign paroxysmal positional vertigo and Meniere's disease (1). Acute unilateral vestibulopathy accounts for 3.2%–9% of consultations in specialized dizziness clinics, with an incidence of 3.5 cases per 100,000 individuals. The mean age at onset ranges from 10 to 50 years. Vestibular dysfunction refers to a series of syndromes resulting from structural or functional abnormalities in the vestibular system (peripheral vestibular organs, vestibular nerves, central vestibular pathways), manifesting as acute or subacute rotational or non-rotational vertigo, with accompanying nausea, vomiting, postural instability and/or oscillopsia. On physical examination, patients typically present with spontaneous nystagmus deviating toward the unaffected side, balance disturbances with a tendency to lean toward the affected side, and a predisposition to falls. Generally, there is no cochlear involvement, with no hearing impairment or tinnitus.

Current international consensus guidelines advocate for combined pharmacological therapy and vestibular rehabilitation training (VRT) as the first-line management. VRT is an individualized, evidence-based, exercise-driven physical therapy approach for patients with vestibular dysfunction that leverages neural plasticity and vestibular compensation mechanisms; through repetitive vestibular stimulation and targeted exercises (including eye movements, postural control and gait training), it expedites central vestibular adaptive reorganization, alleviates vertigo, dizziness and gait instability, reduces fall risk, and restores patients' physical and social functional ability while mitigating associated morbidities (2, 3). VRT should be initiated as early as possible and encompass a comprehensive suite of head and limb exercises specifically tailored to the vestibular system. These exercises include interventions aimed at optimizing the vestibulo-ocular reflex (VOR), vestibulo-spinal reflex (VSR), cervico-ocular reflex, depth perception, somatosensory retraining, gait practice, and aerobic exercise (4). VRT enhances the capacity of the vestibular system to coordinate and control balance by integrating positional sense, visual input, and proprioception. Additionally, it elicits compensatory mechanisms within the central nervous system, facilitates the recovery of vestibular function, and mitigates the risk of chronic vestibular syndrome.

CiteSpace and VOSviewer are Java-based bibliometric analysis software that facilitate rapid mapping of research dynamics and identification of hot spots, frontiers, and trends in scientific literature (5). In this study, we employed CiteSpace and VOSviewer to perform a visual and quantitative analysis of vestibular rehabilitation research on dizziness retrieved from the Web of Science Core Collection and Scopus databases (2014–2024), focusing on research hotspots, leading countries and institutions, highly cited authors, highly cited publications, high-frequency keywords, and keyword burst strength. This analysis aims to identify and track evolving research trajectories, thereby providing theoretical insights for future scientific inquiry in this domain (6) and tracking, thereby providing ideas for future scientific research in this field. The period from 2014 to 2024 was selected because it represents a phase of rapid conceptual and technological evolution in vestibular rehabilitation, including the formalization of persistent postural-perceptual dizziness(PPPD) diagnostic criteria (7), the emergence of virtual reality–based interventions (6, 8), and the increasing emphasis on evidence-based and personalized rehabilitation strategies (3).

## 2 Materials and methods

### 2.1 Data source and collection

**Figure 1:**
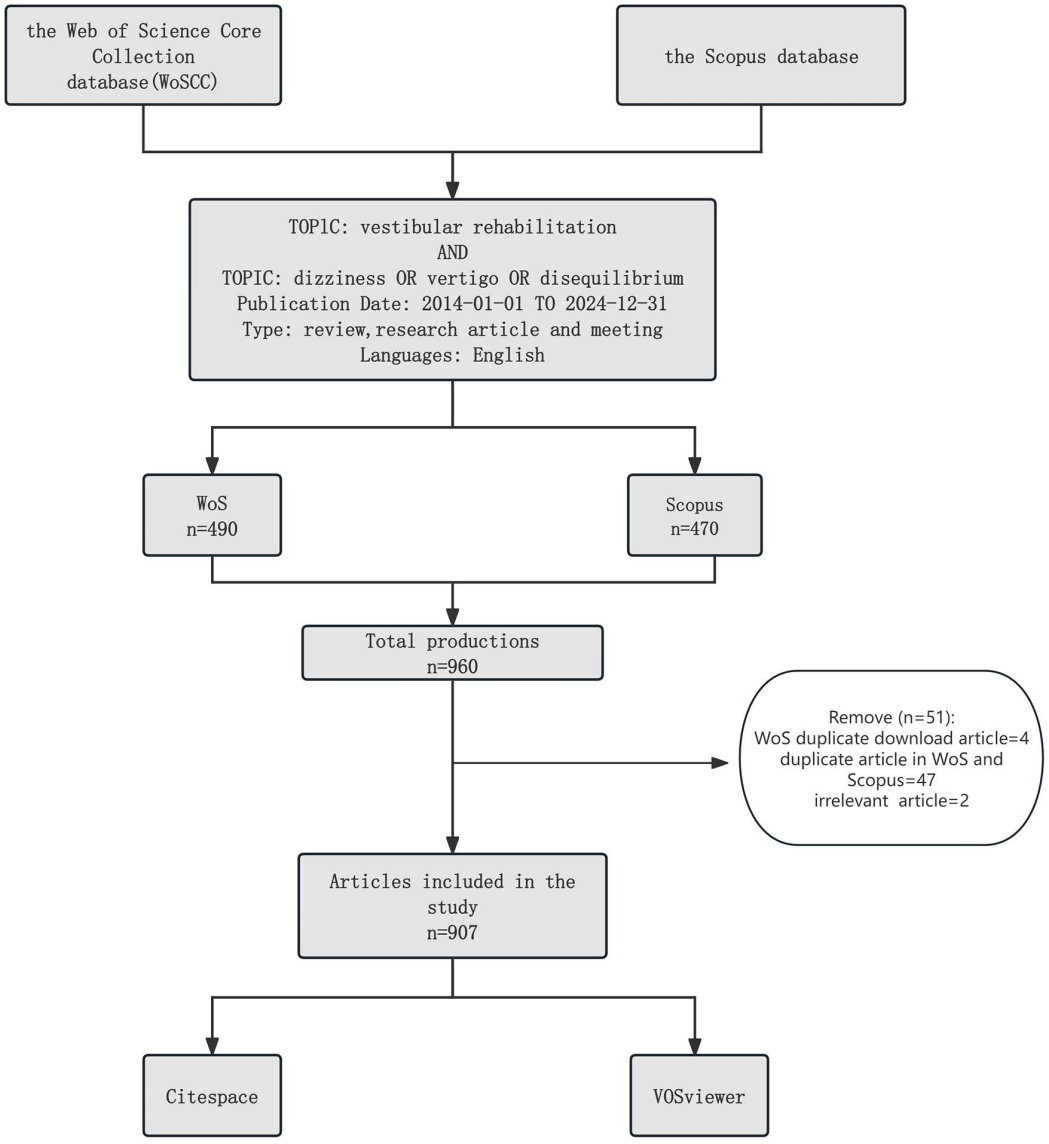
Literature search procedure.

Primary data for the bibliometric analysis were obtained from the Web of Science Core Collection database (WoSCC) and Scopus databases. These databases were selected because they are the two most widely used databases in bibliometric research and provide comprehensive coverage of peer-reviewed literature in medicine, rehabilitation, and allied health sciences. The literature search was conducted on September 21, 2025. The data retrieval strategy is summarized as follows: # 1: TS = (vestibular rehabilitation); #2: TS = ((dizziness) OR (vertigo) OR (disequilibrium)); the ultimate dataset: #1 AND #2. This study included only English-language studies. The time frame for the search period was from January 1, 2014, to December 31, 2024. A literature search was conducted to minimize potential bias from routine database updates on a fixed date. Inclusion criteria were peer-reviewed articles focusing on vestibular rehabilitation. Two researchers from the research group screened the articles based on the inclusion and exclusion criteria. A total of 490 productions were retrieved from the WoSCC database and 470 productions from Scopus database. 51 duplicate articles and 2 invalid articles were excluded. A total of 907 articles were retrieved, including reviews, research articles and meetings (9, 10). Exclusion criteria included conference abstracts, editorials, letters, and studies unrelated to rehabilitation interventions. The literature search procedure is illustrated in Figure 1.

## 2.2 Bibliometric analysis

Publications meeting the inclusion criteria were exported as two plain text files named “download_1-470.csv” from Scopus and “download_1-490.txt” from the WoSCC containing complete records and cited references. Duplicate publications between the Web of Science and Scopus databases were identified and exported in Excel format using R scripts. Duplicate literature was marked and removed from Scopus database using Excel functions. These files were imported into CiteSpace 6.4.R1 and VOSviewer 1.6.20 to construct visual knowledge maps. Concurrently, the documents were exported in tab-delimited file formats and uploaded to an online analysis platform for document metrology with the objective of generating a knowledge map of national/regional collaborative networks.

CiteSpace was configured with the following parameters: time span: January 2014 to December 2024, segmented into 1-year intervals; node types: keywords and references; selection criteria: g-index (k = 25) for each time slice to retain high-impact entities; pruning methods: Pathfinder algorithm, sliced network pruning, and merged network pruning; all other parameters were set to default. VOSviewer was employed with the following parameter settings: association strength was selected as the normalization method. For the purpose of identifying key contributors, minimum publication thresholds were applied to enhance the focus and clarity of the network visualizations: countries (≥8 articles), institutions (≥6 articles), and authors (≥5 articles). In this study, we initially analyzed the publication volume, countries, institutions, authors, and co-cited references. Subsequently, we employed co-occurrence and a cluster map of keywords to identify research hotspots. Finally, we explored research frontiers and trends in this field by examining burst keywords.

## 3 Results

### 3.1 Annual publications and citations

**Figure 2:**
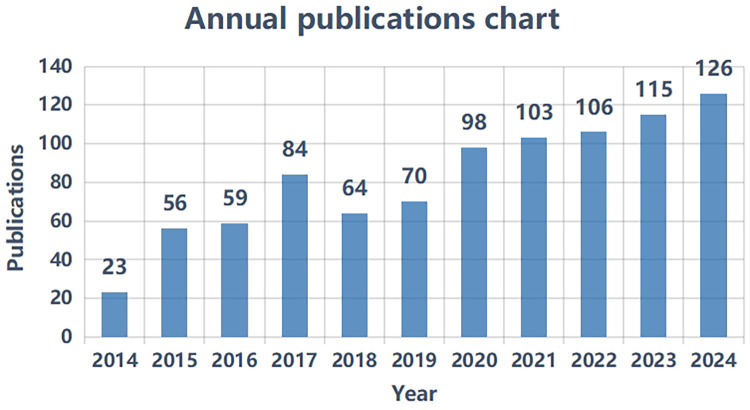
Annual publications trend chart.

Trends in the number of publications can be used to predict future trends in the field. The present study included 907 publications. Figure 2 illustrates the temporal trajectory of the annual publication volume in the field of vestibular rehabilitation research on dizziness from 2014 to 2024. This figure illustrates an overall upward trend. From 23 articles in 2014 to 126 in 2024, the number of publications has shown a steady and rapid growth trend, indicating that over the past 10 years, research interest in the field of vestibular rehabilitation has been continuously rising. From 2014 to 2019, the publication volume gradually increased. This early phase reflects the foundational work being conducted in the field. A moderate increase indicates a steady expansion in research and a corresponding increase in academic attention. During this period, vestibular rehabilitation research on dizziness laid the groundwork for more targeted clinical and experimental investigations. Starting in 2020, the field entered a phase of exponential growth, rising sharply, and peaking in 2024. The substantial surge in publications indicates that vestibular rehabilitation research on dizziness has gained significant traction, attracting an increasing number of researchers and funding.

### 3.2 Distribution of countries/regions

**Figure 3:**
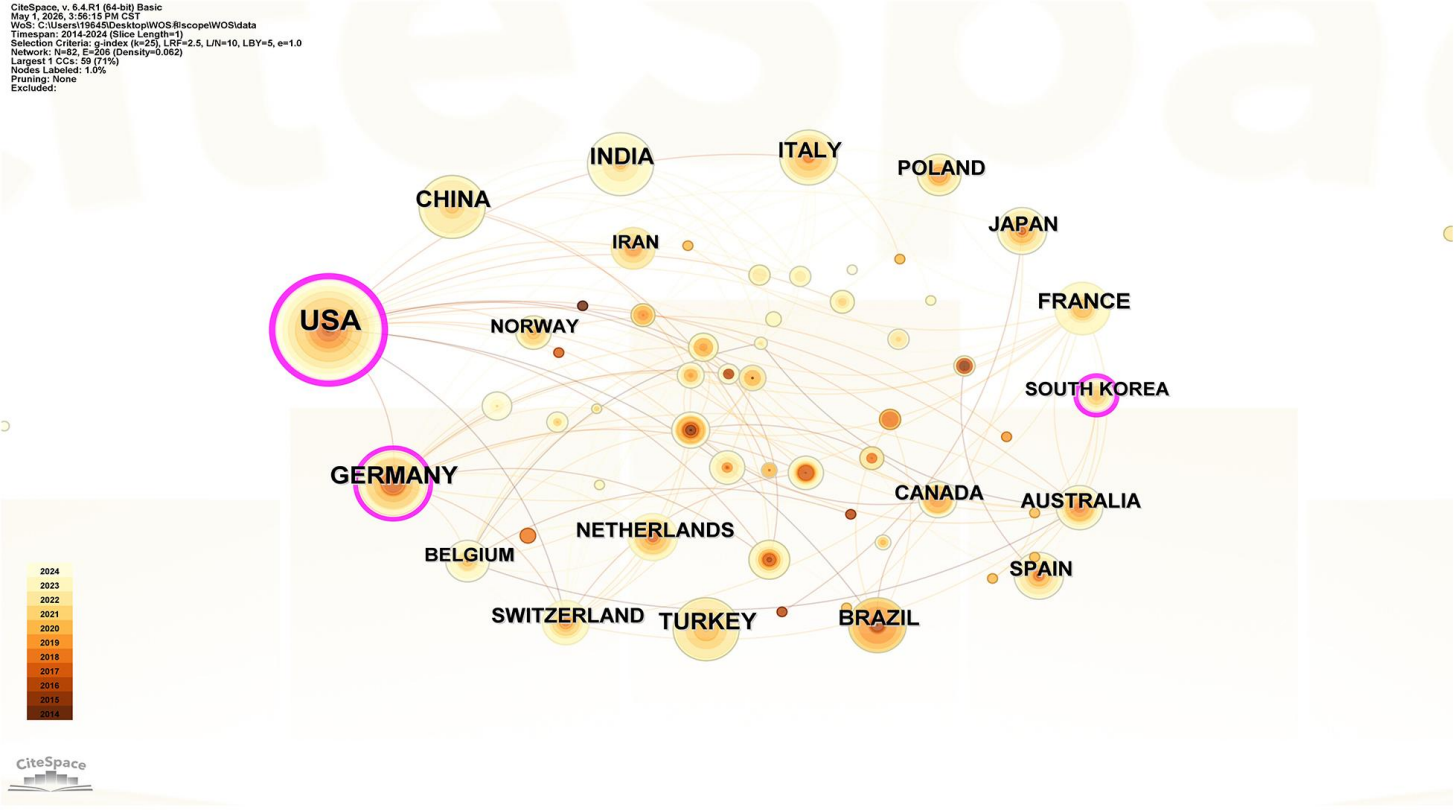
Collaborative network knowledge map of countries/regions.

**Figure 4:**
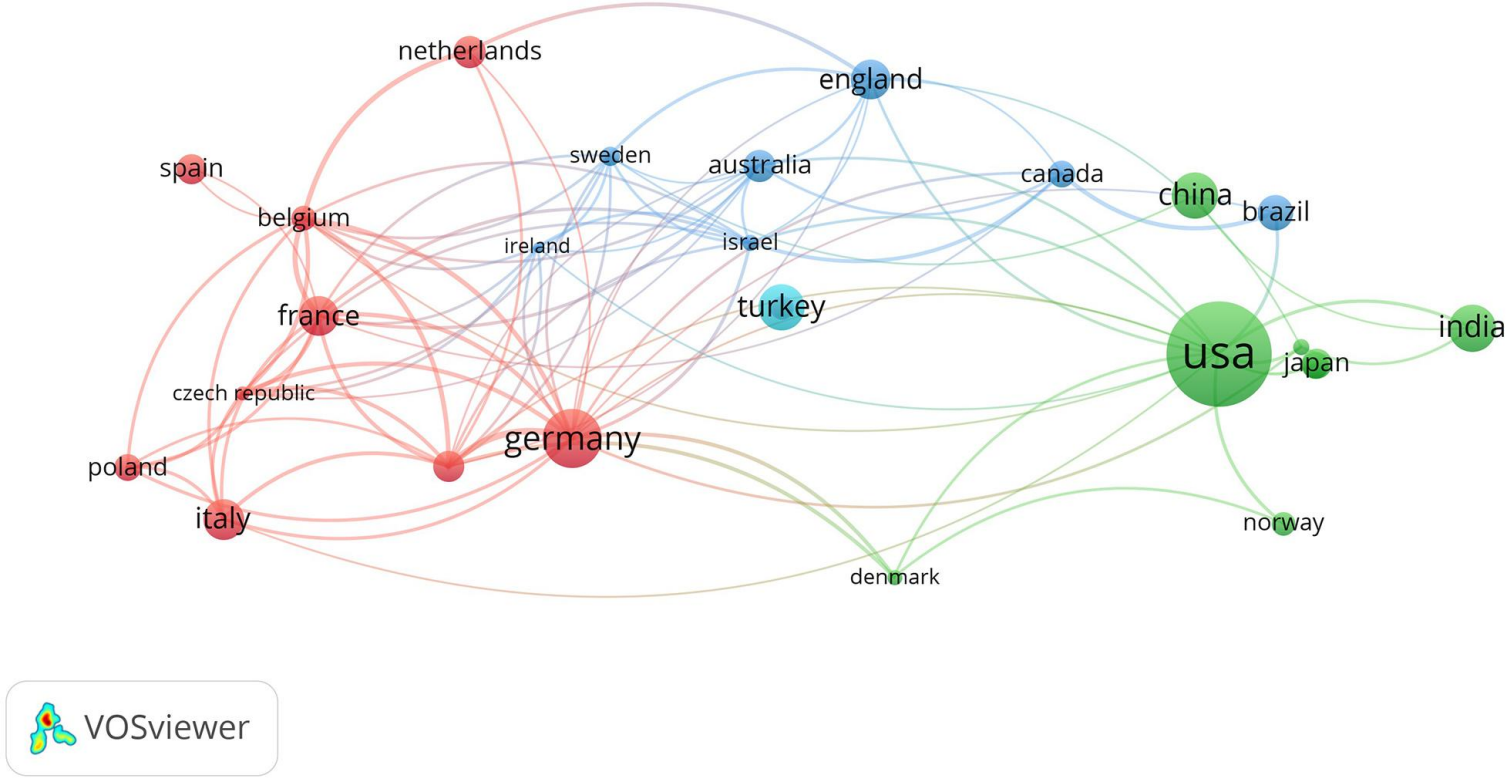
Temporal map of countries collaborative network.

From 2014 to 2024, 93 countries engaged in research on dizziness vestibular rehabilitation, and published relevant literature in this field. In Figure 3, the resulting collaboration map comprises 93 nodes and 325 connecting lines, where each line symbolizes the cooperative relationship between countries. The color of the outer circle of each node represented centrality: countries with darker outer circles exhibited higher centrality, indicating more extensive and influential collaborative relationships. Centrality in co-occurrence networks reflects a node's connective intensity and its capacity to mediate relationships. Highly central nodes generally function as key junctions that integrate different thematic clusters. As illustrated in Table 1, the top five countries were the United States (274 publications), the United Kingdom (83 publications), Germany (76 publications), Canada (52 publications), and China (52 publications) in terms of publication volume. The United States leads in both article output and research centrality. Although its early technological development and in-depth exploration in the field have gained wide international recognition, the higher publication volume from 2014 to 2024 may be primarily attributable to the larger number of VRT researchers based in the US, rather than technological advantages alone. In contrast, China, with a later start, ranks fifth, having published 52 articles, indicating significant growth potential. Figure 4 presents a collaborative network and time overlay graph of countries that have published eight or more articles. The dark color represents before 2019 and the light color represents after 2021. The United States has made significant and long-standing contributions to this field of research, while China, India, and Iran have commenced notable developments in this field.

**Table 1:** Top 10 countries/regions ranked by number of publications.

Rank	Countries/regions	Publications	Centrality
1	UNITED STATES	274	0.2
2	UNITED KINGDOM	83	0.11
3	GERMANY	76	0.19
4	CANADA	52	0.13
5	CHINA	52	0
6	ITALY	42	0.05
7	AUSTRALIA	40	0.03
8	NETHERLANDS	39	0.08
9	SPAIN	39	0
10	BRAZIL	36	0.04

### 3.3 Distribution of institutions

**Figure 5:**
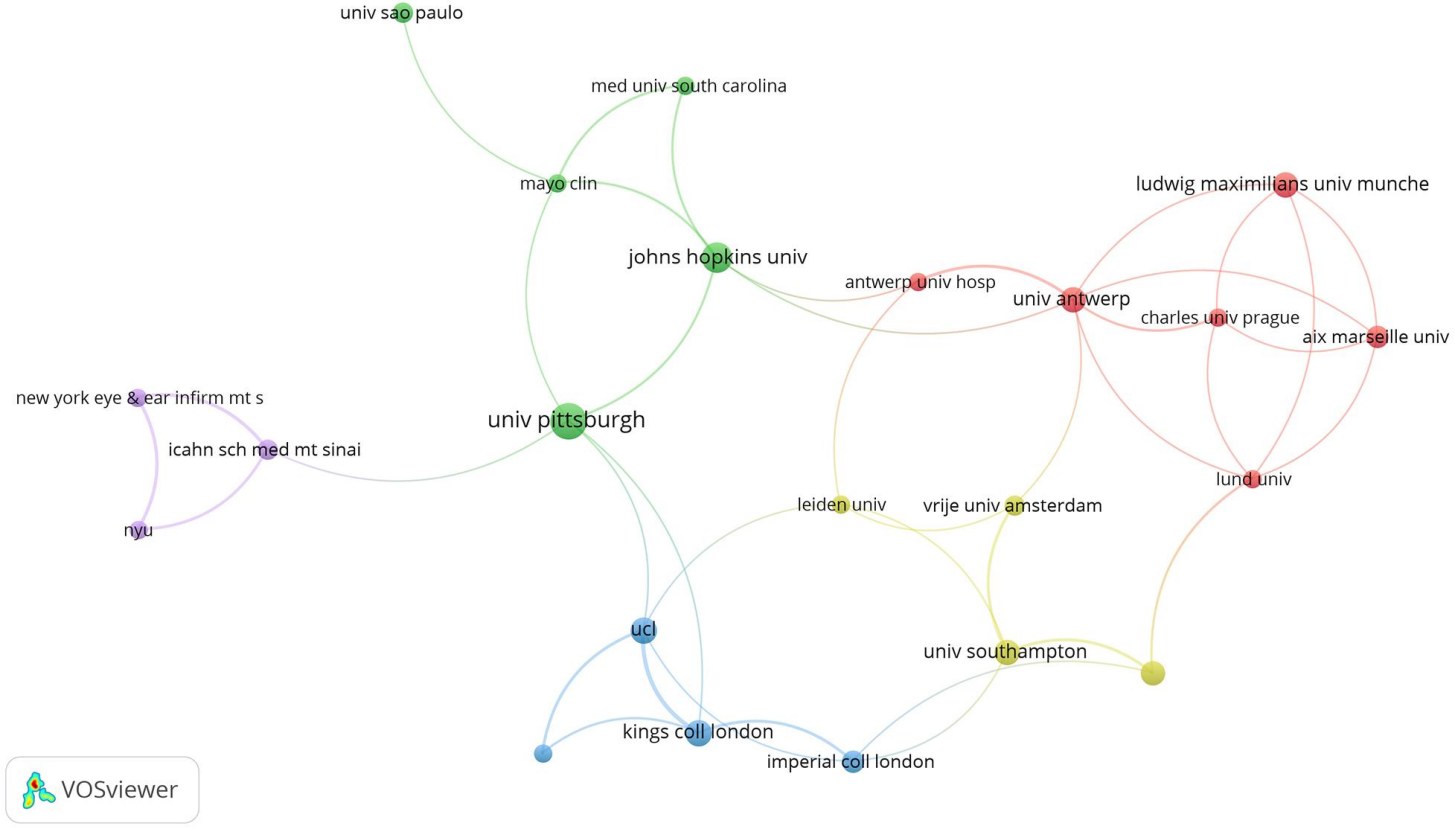
Research institution-based collaborative networks.

**Figure 6:**
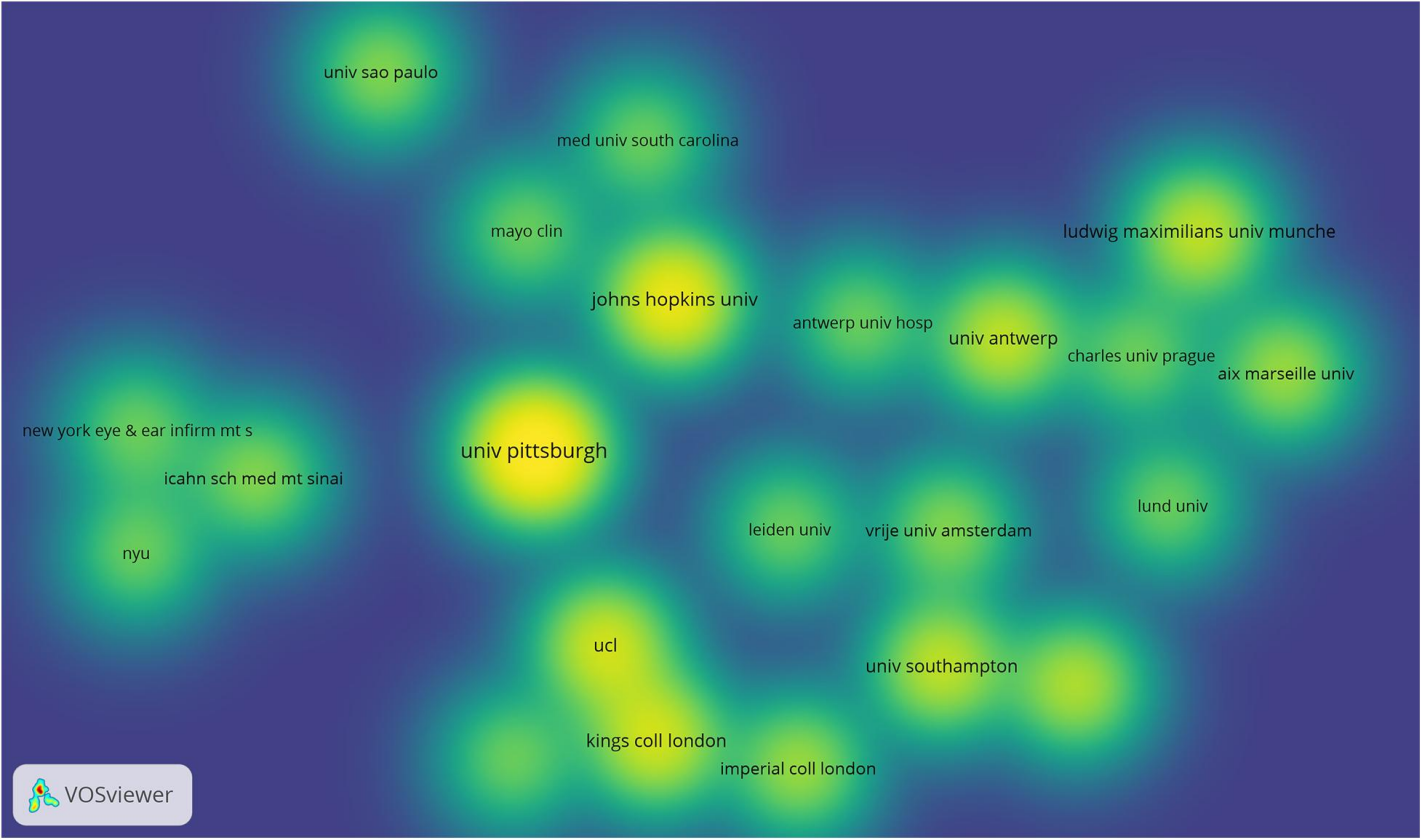
Density of publications by research.

There were 1,556 institutions worldwide, predominantly universities, that conducted research on dizziness vestibular rehabilitation between 2014 and 2024. The collaboration map shows institutions that had published more than six publications. Each node symbolizes an institution, whereas the line symbolizes the cooperative relationship between institutions. When examining the publication outputs of these institutions, significant disparities were observed. As Table 2 shows, the leading pack was the University of Pittsburgh, which contributed 26 research publications to the domain. The Vrije University Amsterdam was closely followed with 19 publications. The third highest institution was the Pennsylvania Commonwealth System of Higher Education, which published 15 research articles. It is worth noting that Johns Hopkins University has the highest centrality (0.09), indicating its core dominant position and pivotal influence in the academic cooperation network of dizziness and vestibular rehabilitation research. As illustrated in Figures 5, 6, these results further reflect the substantial contributions of these key institutions to the theoretical and practical development of dizziness and vestibular rehabilitation.

**Table 2:** Top 10 institutions ranked by number of publications.

Rank	Institutions	Publications	Centrality
1	University of Pittsburgh	26	0.08
2	Vrije University Amsterdam	19	0.03
3	Pennsylvania Commonwealth System of Higher Education (PCSHE)	15	0.01
4	Ludwig-Maximilians-Universität München	13	0.02
5	Klinikum der Universität München	12	0
6	National Hospital for Neurology and Neurosurgery	12	0
7	Johns Hopkins University	12	0.09
8	Imperial College London	11	0.03
9	University Hospitals of Derby and Burton NHS Foundation Trust	11	0
10	University of Oxford Medical Sciences Division	11	0

### 3.4 Analysis of authors

**Figure 7:**
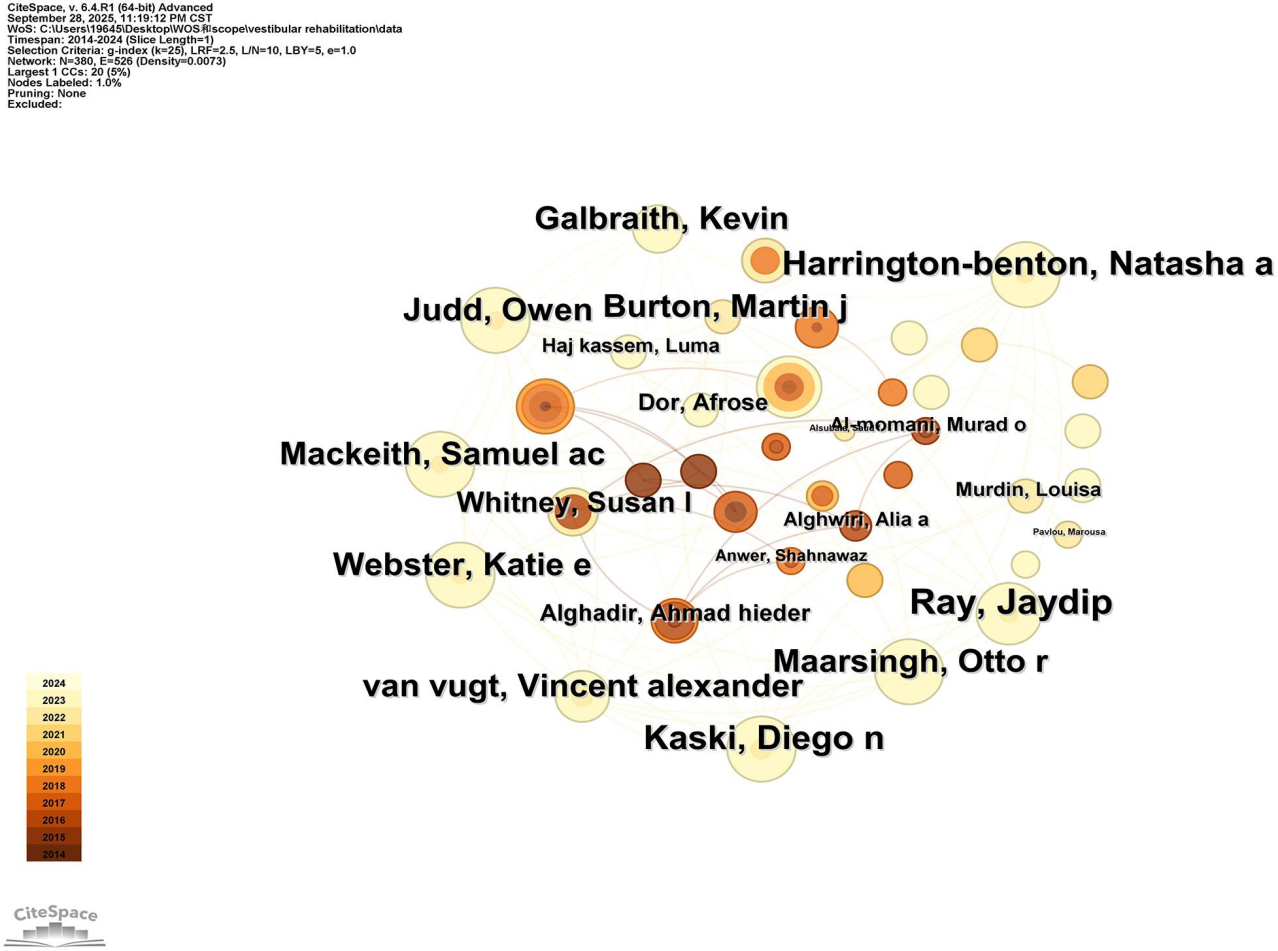
Collaborative network knowledge map of authors.

Figure 7 shows the collaborative network of the authors. As shown in Figure 7, 380 authors contributed to the research on dizziness vestibular rehabilitation from 2014 to 2024. Leading the publication count was Susan L. Whitney from the University of Pittsburgh, USA, with 23 publications, followed by Diego Kaski, an international leader in Vestibular Neuroscience from UCL Queen Square Institute of Neurology (12 publications), and Maarsingh, Otto from Amsterdam Public Health (11 publications). Notably, these authors collectively exhibit low centrality and citation rates.

Table 3 shows that the top 10 most-cited authors during the same period included GP Jacobson from Vanderbilt University (150 citations), Lucy Yardley from the University of Bristol (131 citations), and Susan L. Whitney (124 citations). Among them, although Susan L. Whitney ranks third in citation frequency, she maintains outstanding publication outputs and citation impacts. Her research mainly focuses on balance dysfunction and dizziness symptoms in patients with vestibular disorders. She promotes research innovation by applying virtual reality and augmented reality technologies, aiming to improve the quality of life of patients with inner ear and vestibular diseases (6).

**Table 3:** Top 10 most-cited authors by citation.

Rank	Cited authors	Citations	Centrality
1	Jacobson, Gary P	150	0.05
2	Lucy Yardley	131	0.05
3	Whitney, Susan L	124	0.06
4	Hall, Courtney	111	0.02
5	Herdman, Susan J	109	0.06
6	Neuhauser, Hannelore K	84	0.05
7	Cohen, Helen S	78	0.03
8	Neil Bhattacharyya	68	0.07
9	Michael Strupp	67	0.03
10	Brandt, Thomas	66	0.05

### 3.5 Analysis of cited literature

From 2014 to 2024, 481 references were co-cited in this field, underscoring their collective significance in advancing vestibular rehabilitation for dizziness. Among these, the top four most highly cited publications have played pivotal guiding roles. Hall CD's 2016 publication, *Vestibular Rehabilitation for Peripheral Vestibular Hypofunction: An Evidence-Based Clinical Practice Guideline*, with 56 citations, established a comprehensive framework for evidence-based interventions (3). This guideline meticulously synthesizes existing research, providing clinicians with standardized protocols for assessing and treating peripheral vestibular hypofunction.

The subsequent 2022 update by Hall CD, *Vestibular Rehabilitation for Peripheral Vestibular Hypofunction: An Updated Clinical Practice Guideline From the Academy of Neurologic Physical Therapy of the American Physical Therapy Association*, cited 25 times (2). This iterative approach to guideline development has been instrumental in continuously improving the quality of vestibular rehabilitation, offering practitioners a dynamic reference that can adapt to the evolving landscape of the field.

Staab JP's 2017 publication, *Diagnostic Criteria for Persistent Postural-Perceptual Dizziness (PPPD): Consensus document of the Committee for the Classification of Vestibular Disorders of the Bárány Society*, with 24 citations, filled a critical gap in the diagnostic realm. The introduction of standardized diagnostic criteria for PPPD often involves clear identification (7). This has significantly improved the accuracy of diagnosis, enabling more targeted and effective treatment strategies and has become an indispensable reference for both clinical diagnosis and research studies focused on PPPD.

Finally, Dunlap PM's review article *vestibular rehabilitation: advances in peripheral and central vestibular disorders*, cited 23 times, offered a panoramic view of the latest advancements in the field (11). This review synthesizes cutting-edge research on novel rehabilitation techniques and the integration of advanced technologies, such as virtual reality and augmented reality, into vestibular rehabilitation. By summarizing and analyzing these developments, it served as a roadmap for future research directions, inspiring researchers to explore new avenues for improving patient outcomes and pushing the boundaries of what is possible in vestibular rehabilitation.

### 3.6 Analysis of keywords

#### 3.6.1 Analysis of keyword co-occurrence

**Figure 8:**
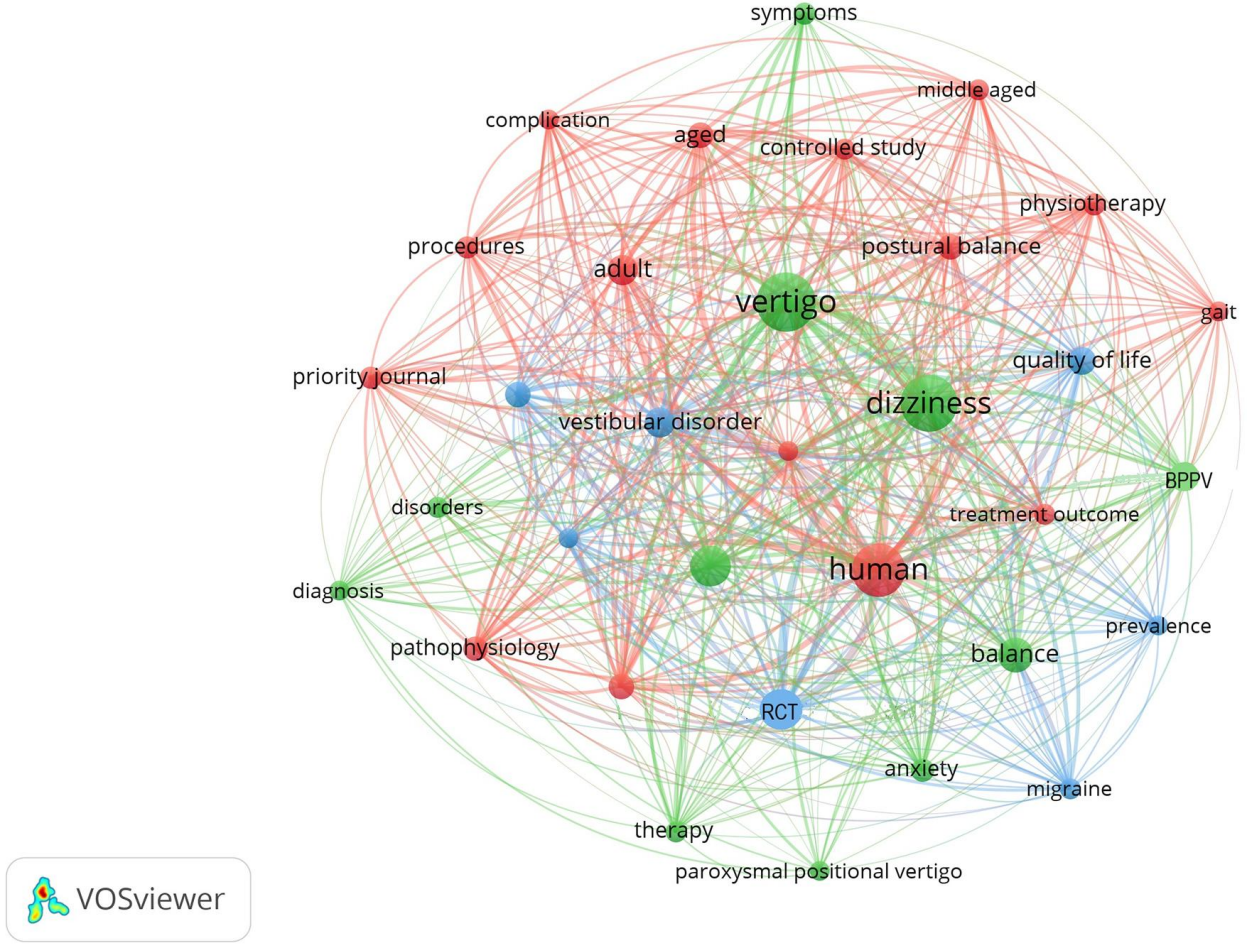
Co-occurrence network knowledge map of keywords.

**Figure 9:**
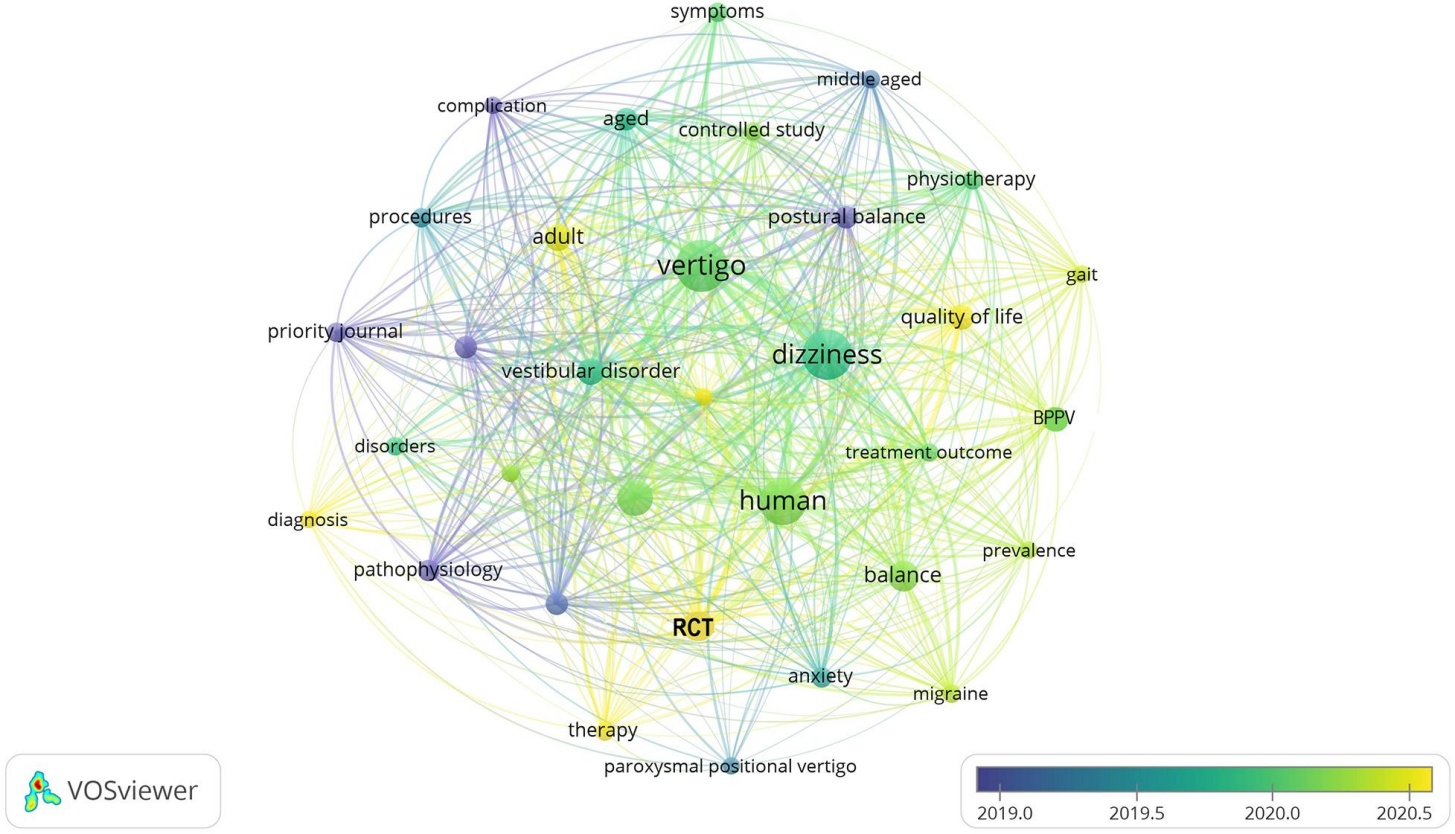
Keyword co-occurrence over time. BPPV, benign paroxysmal positional vertigo; RCT, randomized controlled trial.

Keywords can effectively encapsulate core themes in the relevant literature. The frequency of keyword occurrence reflects the research intensity of the field over a specific period. Higher frequencies indicate greater research prominence. Table 4 shows the top 20 keywords in the field of vestibular rehabilitation for dizziness research from 2014 to 2024. As shown in Figures 8, 9, the keywords establish a systematic academic framework for vestibular rehabilitation research. Dizziness and associated symptoms define the clinical challenge, which is rooted in vestibular dysfunction impairing balance. Vestibular rehabilitation serves as the core intervention and relies on evidence-based protocols to foster vestibuloneural compensation. Balance function acts as a critical endpoint and is quantified via standardized assessments. Quality of life represents the ultimate outcome of integrating physiological and functional recovery. This framework outlines a logical trajectory from pathological mechanisms to intervention design and outcome evaluation. Emerging trends integrate precision medicine and technology for personalized management of chronic dizziness, such as PPPD.

**Table 4:** Top 20 keywords ranked by frequency.

Rank	Keyword	Frequency	Centrality
1	dizziness	239	0.02
2	vertigo	216	0.12
3	vestibular rehabilitation	165	0.01
4	randomized controlled trial (topic)	122	0.19
5	quality of life	106	0.03
6	vestibular disorder	89	0.06
7	rehabilitation	84	0
8	benign paroxysmal positional vertigo	70	0.03
9	aged	64	0.05
10	vestibular diseases	63	0.02
11	balance	63	0.03
12	postural balance	63	0.03
13	body equilibrium	61	0.04
14	pathophysiology	56	0.06
15	procedures	46	0.02
16	symptoms	45	0.08
17	priority journal	43	0.01
18	controlled study	43	0.02
19	anxiety	41	0.02
20	paroxysmal positional vertigo	41	0.02

#### 3.6.2 Analysis of keyword clustering

**Figure 10:**
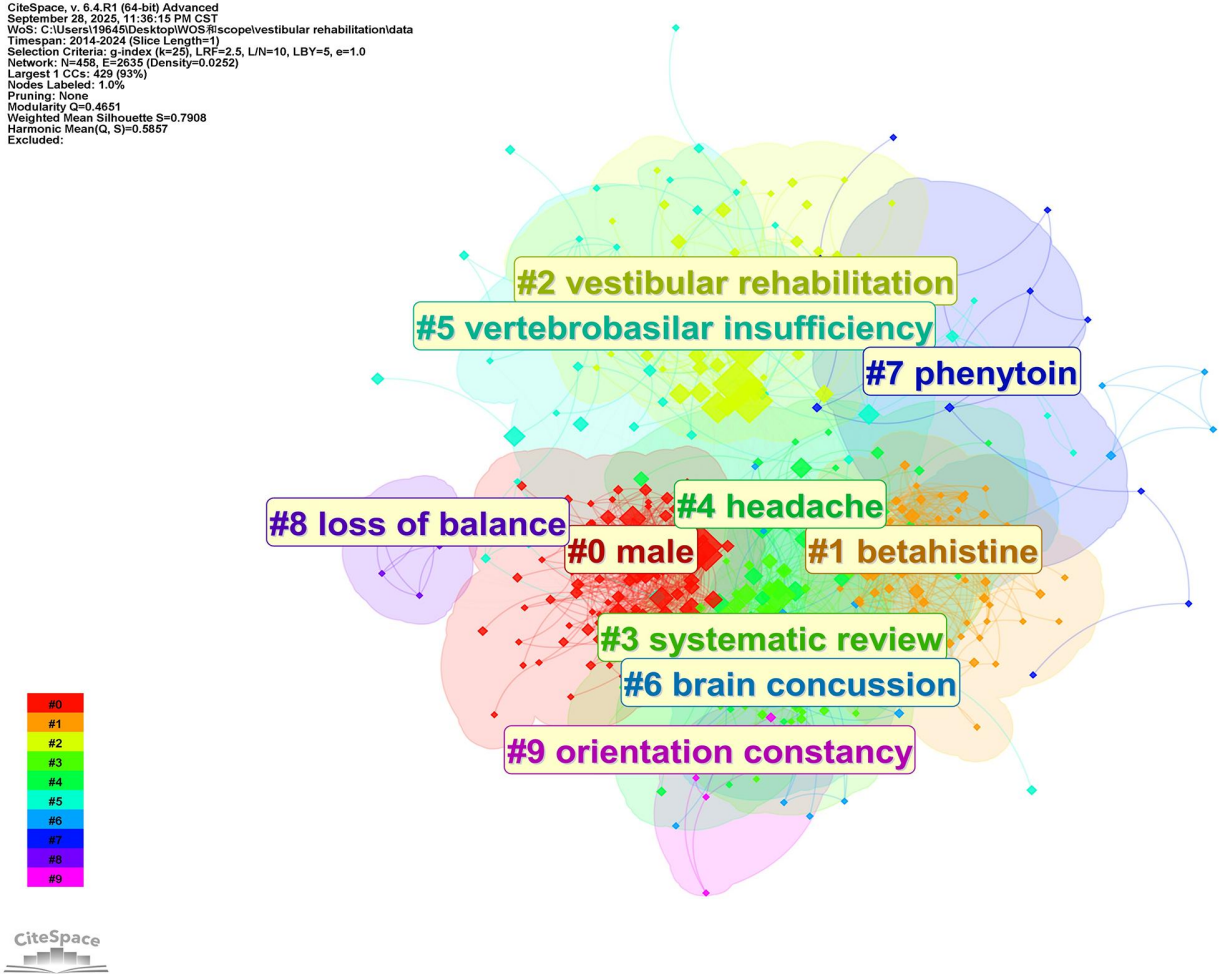
Cluster map of keywords.

Keyword clustering analysis aggregates semantically related keywords into cohesive thematic clusters. A lower cluster number indicates higher thematic concentration, signifying that fewer clusters subsume a greater density of interconnected keywords. This metric reflects the degree of conceptual convergence and interdisciplinary integration within a field, with compact clustering often denoting mature research paradigms. As shown in Figure 10, the top six clusters with corresponding profile values were #0 male, #1 betahistine, #2 vestibular rehabilitation, #3 systematic review, #4 headache, #5 vertebrobasilar insufficiency, #6 brain concussion, #7 phenytoin, #8 loss of balance, and #9 orientation constancy. These keywords collectively reflect the fact that current research focuses on vestibular function, encompassing research subjects, core interventions, methodologies, and associated disorders. These include specific populations, such as “males”, indicating attention to gender-specific vestibular issues and core disorders—vestibular dysfunction and related symptoms such as “loss of balance” and “orientation constancy”, which are key vestibular indicators, alongside linked conditions such as “headache”, “concussion” and “vertebrobasilar insufficiency”. Interventions cover “vestibular rehabilitation”, which is a core non-pharmacological approach and drugs, spanning pharmacological and non-pharmacological treatments. Additionally, a “systematic review” highlighted a focus on integrating evidence to strengthen evidence-based support for diagnosing, treating, and rehabilitating vestibular disorders.

#### 3.6.3 Analysis of keyword burst

**Figure 11:**
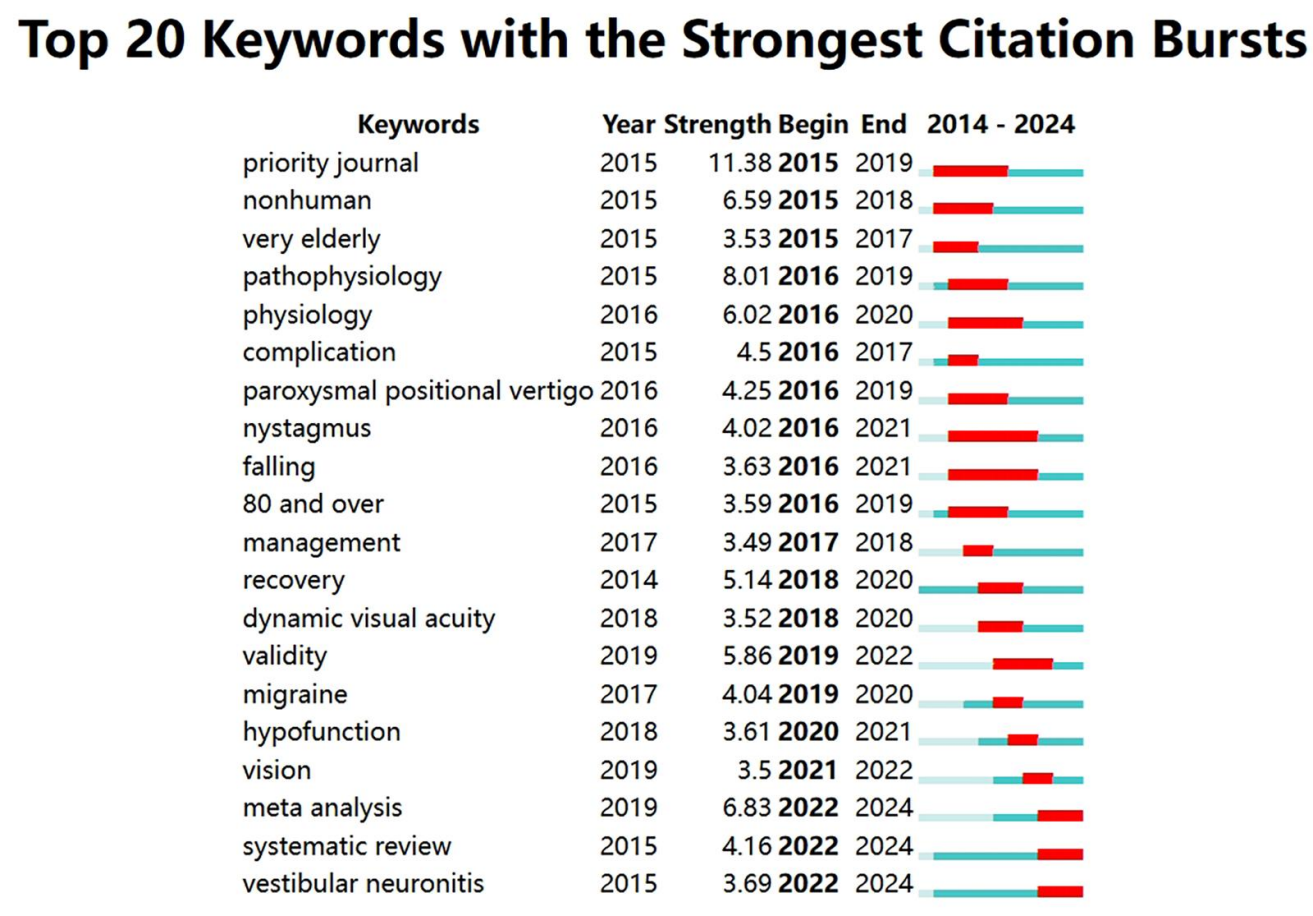
Top 20 keywords with the strongest citation bursts.

Keyword emergence analysis involves identifying words with abrupt proliferation within a defined timeframe, a method used to elucidate research evolution trends, mapping timelines for predicting technological frontiers, pinpointing emerging research hotspots, and forecasting future research directions. In visualizations, the strength metric quantifies the intensity of keyword bursts, reflecting their disciplinary impact and temporal salience. Figure 11 shows the top 20 keywords. The earliest detected burst corresponds to “pathophysiology”, which also exhibits a longer sustained burst period, demonstrating a deeper study of the pathology of the disease. The keyword “vestibular neuronitis” still experiences bursts.

## 4 Discussion

### 4.1 Research status

Vestibular rehabilitation therapy (VRT) is a systematic intervention based on canalith repositioning, vestibular adaptation, habituation training, and sensory substitution. Relying on neuroplastic reorganization, VRT enhances patients' ability to coordinate vestibular, visual, and proprioceptive inputs for balance control, thereby promoting dynamic compensation (7, 11). Tailored to individual pathologies, VRT protocols typically include gaze stability exercises (X1 viewing and X2 viewing), habituation training to reduce vestibular hypersensitivity, balance and gait retraining, and endurance conditioning.

Numerous studies have demonstrated that VRT combined with pharmacotherapy yields superior outcomes compared with mono-pharmaceutical interventions, with sustained improvement in dizziness symptoms. Early initiation of VRT within two weeks of symptom onset is critical for optimizing therapeutic outcomes (12–15).

Consistent with previous bibliometric reviews, the present study adopted CiteSpace to map the global research landscape of vestibular rehabilitation for dizziness, systematically analyzing research hotspots, core authors, high-output countries/institutions, and collaborative networks. Compared with existing published literature, our findings further quantify the global research layout and fill the gap in macroscopic trend analysis over the 2014–2024 period.

Centrality is a key index to evaluate a node's position and influence in the VRT cooperation network. Higher centrality indicates a core hub role with strong resource integration and academic radiation capacity, while lower centrality means a peripheral position with limited cooperative connections and information access efficiency, reflecting the imbalance of network development.

1. At the national level, the United States demonstrates the highest research output and intermediary centrality, enjoying substantial international recognition. This can be attributed to the early initiation of vestibular rehabilitation research in Europe and America coupled with in-depth institutional collaboration. In contrast, research in China started relatively late, with scattered institutional cooperation and insufficient cross-regional academic exchange, indicating considerable room for progress in future research layout and collaborative construction.
2. At the author level, the collaborative network centered on Whitney in the field of vestibular rehabilitation is still evolving, with tighter collaboration among international authors. It is suggested that Chinese scholars strengthen internal academic communication and establish stable cooperative ties with international teams to accelerate the innovation and transformation of vestibular rehabilitation research.
3. Keyword co-occurrence clustering analysis revealed that beyond traditional investigations into vestibular rehabilitation for various dizziness disorders, the academic community has expanded its scope to include applications such as vestibular migraine and post-craniocerebral trauma rehabilitation. Concurrently, keyword burst analysis demonstrated a research trajectory shifting from broad definitions of dizziness to disease-specific classification, transitioning from symptomatic relief to balance function improvement, and ultimately toward personalized vestibular rehabilitation strategies.

### 4.2 Development direction

This summary of vestibular rehabilitation research hotspots reveals a paradigm shift in academic inquiry, where investigations have transcended basic neurophysiological mechanisms to address complex clinical entities. Notable expansions include targeted research on vestibular migraines, traumatic brain injuries, and post-concussion syndromes. Concurrently, disciplinary integration has evolved from isolated clinical practices to systematic cross-disciplinary collaboration: neurologists contribute central nervous system plasticity insights, otorhinolaryngologists address peripheral vestibular pathologies, rehabilitation specialists design functional training protocols, and psychologists manage associated anxiety disorders (16). Notably, traditional acupuncture and complementary therapy have been increasingly integrated into mainstream evidence-based rehabilitation; existing studies have confirmed that acupuncture can regulate vestibular neural excitability and assist functional compensation, providing new ideas for integrated rehabilitation models (17, 18).

Technological advancements, particularly artificial intelligence (19), are emerging as a promising direction in the field. While AI-based simulations have shown potential in enabling precise assessment and targeted training of vestibular function, with preliminary evidence suggesting improvements in the precision, engagement, and efficacy of rehabilitation protocols, this application remains an emerging area of study that has not yet been widely used or accepted in clinical practice.

### 4.3 Evolution of vestibular rehabilitation research from 2014 to 2024

Over the past decade (2014–2024), VRT research has undergone a remarkable evolution, characterized by expanding global participation, refined therapeutic mechanisms, and emerging disease-centric focuses. Bibliometric analyses of international databases have delineated the quantitative and qualitative shifts in this field, revealing key trends in publication output, regional contributions, and research hotspots.

Thematic evolution has been equally prominent. In the early phase (2014–2018), research primarily focused on validating the efficacy of traditional VRT techniques and elucidating basic vestibular compensation mechanisms (20). The “cerebellar inhibition theory” was gradually challenged by neuroimaging evidence, with functional MRI studies revealing asymmetric cerebral activation patterns post-vestibular injury and supporting the role of neuronal synaptic plasticity, ion channel regulation, and sensory integration in static and dynamic compensation (21).

From 2019 to 2024, the field witnessed two critical shifts: the rise of disease-specific VR strategies and the exploration of post-infectious vestibular disorders. Research expanded to encompass vestibular schwannoma, vestibular migraine, and COVID-19-associated vestibular neuritis, with case reports and cohort studies highlighting viral infection as a novel etiological factor. Concurrently, the validation of diagnostic tools such as the Head Impulse, Nystagmus, Test of Skew (HINTS) for stroke-associated acute vestibular syndrome became a consensus-building topic, as its superior sensitivity than early MRI in detecting stroke-related vertigo was widely corroborated by co-citation analyses (22, 23).

### 4.4 Evidence-based therapeutic recommendations identified from bibliometric trends

Bibliometric trends have distilled evidence-based therapeutic recommendations that align with disease pathology, intervention timing, and treatment modality. These recommendations are stratified by disorder type and supported by meta-analyses, systematic reviews, and high-quality clinical studies, addressing both peripheral and central vestibular disorders.

For peripheral acute vestibular syndrome (pAVS), including vestibular neuritis, recent meta-analyses strongly support early VRT combined with corticosteroids. A 2024 systematic review and meta-analysis of 5 RCTs (235 patients) demonstrated that initiating VRT within 2 weeks of onset, alongside corticosteroids, significantly improved Dizziness Handicap Inventory (DHI) scores at 1-month (*P* = .00) and 12-month (*P* *=* .01) follow-up follow-ups compared to corticosteroids alone, with no reported adverse effects (24). For vestibular neuritis specifically, a separate meta-analysis of 12 RCTs (536 patients) confirmed that combined VRT-corticosteroid therapy enhanced quality of life and accelerated canal paresis recovery more effectively than monotherapy, offering a safe alternative for patients unable to tolerate corticosteroids (25).

In BPPV, the most prevalent peripheral vestibular disorder, canalith repositioning maneuvers remain first-line, with evidence indicating superior efficacy to pharmacotherapy or generic exercise programs (26). However, integrative approaches have gained traction in Chinese clinical practice, where VRT combined with Epley maneuvers, Gastrodia elata, or betahistine has shown incremental benefits in symptom resolution and recurrence prevention (27). For chronic balance disorders without overt vestibular pathology, optokinetic stimulation (OKS) emerges as a promising adjunct, with meta-analyses showing significant improvements (28).

Notably, current evidence highlights gaps in intervention standardization. Despite guideline recommendations for combined VRT-physical therapy, most studies suffer from low evidence levels, small sample sizes, and heterogeneous protocols, emphasizing the need for standardized outcome measures and large-scale RCTs to validate emerging therapies.

### 4.5 Clinical implications and future directions

The evolution of virtual reality research over the past decade translates to actionable clinical implications and identifies critical avenues for future investigation. Clinically, the shift toward personalized, disease-centric interventions necessitates interdisciplinary collaboration between otolaryngologists, neurologists, and physiotherapists to optimize diagnosis-driven VRT planning (29).

Three key clinical implications can be concluded based on the identified research hotspots and published evidence: (1) Timing of intervention is paramount—early VRT (within 2 weeks for pAVS, 72 hours for acute vestibular injury) should be prioritized to leverage neuroplastic potential and prevent chronic vestibular syndrome (8, 30). (2) Multimodal approaches outperform monotherapy; for example, combining VRT with corticosteroids in neuritis, manual reduction in BPPV, or acupuncture in age-related vertigo addresses complex pathophysiological mechanisms (31). (3) Individualized scheme for elderly population. Geriatric vestibular dysfunction is often complicated with sensory degeneration and high fall risk; rehabilitation schemes should integrate balance training, fall prevention, and psychological intervention (32).

Future research directions must address existing evidence gaps and capitalize on technological and mechanistic advances. First, large-scale, multicenter RCTs are urgently needed to validate integrative therapies, OKS, and post-COVID vestibular rehabilitation, with standardized protocols to enable meta-analytic synthesis. Second, mechanistic studies should explore the interplay between neuroplasticity, gut-brain-axis, and inflammatory pathways in vestibular compensation, leveraging advanced imaging (e.g., 18F-FDG PET) and molecular techniques to identify novel therapeutic targets (33). Third, technological innovation—including virtual reality-based simulation training, wearable balance monitors, and AI-driven personalized exercise algorithms—holds promise to enhance treatment adherence and objective outcome tracking (8, 34, 35).

Additionally, global harmonization of research methodologies is critical to reconcile regional differences in treatment paradigms (36). Comparative effectiveness studies between Western and integrative VRT approaches could identify universal best practices (11), while cross-cultural studies may elucidate sociocultural factors influencing treatment outcomes. Addressing the high economic burden of falls through Virtual Reality-based fall prevention programs for the elderly represents a pressing public health priority (37).

Finally, educational initiatives are needed to standardize VRT for clinicians, ensuring that evidence-based techniques (e.g., HINTS for diagnosis, personalized multisensory training) are translated into clinical practice. This includes training physiotherapists to assess sensory dependence and tailor interventions to individual deficits, reducing the unspecific use of VRT in dizzy patients.

## 5 Limitation

This study has several limitations. First, this bibliometric analysis was confined to data derived solely from the Web of Science Core Collection (WoSCC) and Scopus databases, excluding alternative databases. This methodological choice may have failed to capture relevant studies indexed exclusively in other databases, potentially underrepresenting critical research and thus compromising the comprehensiveness of the findings. Second, the exclusive focus on English-language publications inherently overlooks non-English contributions, particularly from non-Anglophone regions. Such a limitation could skew the representation of global research trends in vestibular rehabilitation for dizziness, as it may not adequately reflect regional advancements or cross-linguistic research divergence. These constraints highlight the need for future studies to adopt multi-database integration and multilingual inclusion to enhance the generalizability of the bibliometric insights. Third, this study only included publications published in the recent 10 years; therefore, it cannot reflect the long-term evolutionary trajectory of this research field, which constitutes a limitation of the time-span selection in this study.

## 6 Conclusion

This bibliometric analysis revealed that annual publications in vestibular rehabilitation for dizziness have exhibited a steady upward trend from 2014 to 2024, reflecting a growing interdisciplinary interest. Key research includes unilateral vestibular hypofunction, Ménière's disease, vestibular migraine, posttraumatic vestibulopathy, and balance control mechanisms (38). Recent studies indicate a shift toward complex disorders, with emerging investigations into vestibular rehabilitation for post-concussion syndrome and neurodegenerative diseases. Despite these advances, critical research gaps remain: first, the clinical evidence base for pediatric vestibular disorders is underdeveloped, lacking standardized assessment protocols and targeted rehabilitation strategies tailored to immature vestibular systems; second, geriatric vestibular dysfunction is rarely integrated into holistic fall prevention frameworks, with insufficient exploration of age-specific neuroplastic potential and rehabilitative responsiveness; third, the application of AI technologies from experimental research to regular clinical use is restricted by inadequate validation in diverse patient groups and unclear practical implementation methods.

Future research is needed to expand clinical applications to pediatric vestibular disorders and geriatric fall prevention, and leverage AI-driven innovations, such as machine learning-based balance assessment tools and virtual reality-enhanced neuroplasticity training. Integrating AI with sensor technology and rehabilitation medicine will advance data-driven, patient-specific interventions, and optimize functional outcomes and care personalization.

## Data Availability

The original contributions presented in the study are included in the article/Supplementary Material, further inquiries can be directed to the corresponding author.
